# Expert Consensus on Subxiphoid and Subcostal Arch Thoracoscopic Resection for the Treatment of Thymoma

**DOI:** 10.1111/1759-7714.70094

**Published:** 2025-06-26

**Authors:** Xunliang Yin, Hongtao Duan, Zhengwei Zhao, Xiuyi Zhi, Gening Jiang, Shugeng Gao, Chun Chen, Haiquan Chen, Xiaofei Li, Jian Hu, Lanjun Zhang, Hecheng Li, Zhentao Yu, Hui Tian, Zhenfa Zhang, Q. I. Xue, Yunchao Huang, Junqiang Fan, Qing Geng, Dong Chao, Taiqian Gong, Wenjie Jiao, Zhigang Li, Hongjing Jiang, Yufeng Ba, Jigang Dai, Bo Deng, Shuchen Chen, Shouyin Di, Jianyong Ding, Liang Duan, Jiang Fan, Yingtong Feng, Yunjiu Gou, Zhitao Gu, Yong Han, Jinxi He, Xiaomin He, Jianbin Hou, Feng Jiang, Bin Li, Qingxin Li, Shuben Li, Xiangnan Li, Chunguang Li, Zhongcheng Li, Jianhong Lian, Junguo Liang, Naixin Liang, Jiangbo Lin, Dazhi Liu, Huifeng Liu, Junfeng Liu, Wei Liu, Yangchun Liu, Shumin Wang, Shijie Zhang, Shaohua Ma, Nan Wu, Wenhao Liu, Jun Yi, Chenxi Zhong, Songtao Xu, Shengmao Ma, Hui Sun, Hai Xu, Li Wei, Qiang Pu, Ruixiang Zhang, Wenxiang Wang, Desong Yang, Jiufa Wu, Nan Sun, Ping Xu, Haitao Ma, Lei Xue, Shunkai Zhou, Haidong Wang, Enwu Xu, Guangxia Wei, Kun Wang, Guofang Zhao, Zhongmin Peng, Guoqiang Yin, Xueliang Yang, Teng Mao, Deping Zhao, Hua Tang, Jiandong Mei, Yun Wang, Weiguo Jin, Dongsheng Yue, Hao Zhang, Liwei Zhang, Dianbo Li, Yongfu Ma, Saibo Pan, Luming Wang, Ming Wu, Zixiang Wu, Yanhe Su, Yu Qi, Jianjun Qin, Hongcan Shi, Guangqiang Zhao, Fengwei Tan, Tianhu Wang, Han Yang, Haoxian Yang, Yingguo Zhang, Kai Cui, Wuping Wang, Wenhai Li, Ting Chang, Kai Guo, Yawei Dou, Shudong Li, Dong Han, Kunxiang Gao, Chao Ding, Yong Zhang, Guangjian Zhang, Junke Fu, Wen Liu, Qingchun Liu, Xi Zhang, Weidong Lv, Jia Liu, Guangyan Lei, Yunfeng Zhang, Tao Xu, Yong Zhang, Xiaoquan Ding, Hongtao Tang, Xiaoping Liang, Xiaokang Zhang, Peng Ge, Junqi Wang, Jiantao Jiang, Jianzhong Li, Ruibin Xu, Yize Guo, Mingliang Xing, Jiakuan Chen, Honggang Liu, Ende Yang, Shaoyi Cheng, Liping Tong, Haihua Guo, Tao Wang, Guang Yang, Jianyong Sun, Yongshi Liu, Nianlin Xie, Lei Wang, Feng Tian, Daixing Zhong, Yunfeng Ni, Jian Wang, Jinbo Zhao, Jie Lei, Lijun Huang, Zhongping Gu, Tao Zhang, Xiaoping Wang, Tao Jiang, Qiang Lu, Xiaolong Yan, Yongan Zhou

**Affiliations:** ^1^ Air Force Medical University Tangdu Hospital, Thoracic Surgery Xi'an China; ^2^ Xuanwu Hospital Capital Medical University, Thoracic Surgery Beijing China; ^3^ Tongji University Affiliated Shanghai Pulmonary Hospital, Department of Tuberculosis, Thoracic Surgery Wuhan China; ^4^ Chinese Academy of Medical Sciences and Peking Union Medical College, Thoracic Surgery Beijing China; ^5^ Fujian Medical University Union Hospital, Thoracic Surgery Fuzhou China; ^6^ Fudan University Shanghai Cancer Center, Thoracic Surgery Shanghai China; ^7^ Xi'an International Medical Center Hospital, Thoracic Surgery Xi'an China; ^8^ The First Affiliated Hospital of Zhejiang University School of Medicine, Thoracic Surgery Hangzhou China; ^9^ Sun Yat‐Sen University Cancer Center, Thoracic Surgery Guangzhou China; ^10^ Ruijin Hospital, Thoracic Surgery Shanghai China; ^11^ The First Affiliated Hospital of Shandong First Medical University, Thoracic Surgery Jinan China; ^12^ Tianjin Medical University Cancer Institute & Hospital, Thoracic Surgery Tianjin China; ^13^ Yunnan Cancer Hospital, Thoracic Surgery Kunming China; ^14^ The Second Affiliated Hospital of Zhejiang University School of Medicine Jiande Hospital, Thoracic Surgery Hangzhou China; ^15^ Wuhan University People's Hospital, Thoracic Surgery Wuhan China; ^16^ 940th Hospital of Joint Logistics Support Force of Chinese PLA, Thoracic Surgery Lanzhou China; ^17^ Sixth Medical Center of PLA General Hospital, Thoracic Surgery Beijing China; ^18^ The Affiliated Hospital of Qingdao University, Thoracic Surgery Qingdao China; ^19^ Shanghai Chest Hospital, Thoracic Surgery Shanghai China; ^20^ Henan Cancer Hospital Affiliated Cancer Hospital of Zhengzhou University, Thoracic Surgery Zhengzhou China; ^21^ Army Medical University Xinqiao Hospital, Thoracic Surgery Chongqing China; ^22^ Third Military Medical University Second Affiliated Hospital, Thoracic Surgery Chongqing China; ^23^ Chinese PLA General Hospital, Thoracic Surgery Beijing China; ^24^ Fudan University Zhongshan Hospital, Thoracic Surgery Shanghai China; ^25^ Shanghai First People's Hospital, Thoracic Surgery Shanghai China; ^26^ Huaihai Hospital Affiliated to Xuzhou Medical University, Thoracic Surgery Xuzhou China; ^27^ Gansu Province People's Hospital, Thoracic Surgery Lanzhou China; ^28^ Shanghai Jiao Tong University School of Medicine Shanghai Chest Hospital, Thoracic Surgery Shanghai China; ^29^ Air Characteristic Medical Center Affiliated to PLA Air Force Military Medical University, Thoracic Surgery Beijing China; ^30^ General Hospital of Ningxia Medical University, Thoracic Surgery Yinchuan China; ^31^ Shanghai Children's Medical Center Affiliated to Shanghai Jiaotong University School of Medicine, Thoracic Surgery Shanghai China; ^32^ Anyang Tumor Hospital, Thoracic Surgery Anyang China; ^33^ Jiangsu Cancer Hospital, Thoracic Surgery Nanjing China; ^34^ Lanzhou University Second Hospital, Thoracic Surgery Lanzhou China; ^35^ First Affiliated Hospital of Guangzhou Medical University, Thoracic Surgery Guangzhou China; ^36^ The First Affiliated Hospital of Zhengzhou University, Thoracic Surgery Zhengzhou China; ^37^ Qinghai University Affiliated Hospital, Thoracic Surgery Xining China; ^38^ Shanxi Cancer Hospital, Thoracic Surgery Taiyuan China; ^39^ The Affiliated Hospital of Inner Mongolia Medical University, Thoracic Surgery Hohhot China; ^40^ Peking Union Medical College Hospital, Thoracic Surgery Beijing China; ^41^ General Hospital of Northern Theater Command Department of Surgery, Thoracic Surgery Shenyang China; ^42^ The Eighth Medical Center of Chinese PLA General Hospital, Thoracic Surgery Beijing China; ^43^ The Fourth Affiliated Hospital of Hebei Medical University, Thoracic Surgery Shijiazhuang China; ^44^ The First Hospital of Jilin University, Thoracic Surgery Changchun China; ^45^ Jiangxi Provincial People's Hospital, Thoracic Surgery Nanchang China; ^46^ General Hospital of Northern Military Area, Thoracic Surgery Shenyang China; ^47^ Peking University First Hospital, Thoracic Surgery Beijing China; ^48^ Peking University Cancer Hospital, Thoracic Surgery Beijing China; ^49^ General Hospital of Eastern Theatre Command, Thoracic Surgery Nanjing China; ^50^ People's Hospital of Ningxia Hui Autonomous Region, Thoracic Surgery Yinchuan China; ^51^ Ganzhou People's Hospital, Thoracic Surgery Ganzhou China; ^52^ Harbin Medical University Cancer Hospital, Thoracic Surgery Harbin China; ^53^ Hunan Medical University, Thoracic Surgery Changsha China; ^54^ West China Hospital of Medicine, Thoracic Surgery Chengdu China; ^55^ Hunan Cancer Hospital, Thoracic Surgery Changsha China; ^56^ Jiangxi Cancer Hospital, Thoracic Surgery Nanchang China; ^57^ Liaoning Cancer Hospital and Institute, Thoracic Surgery Shenyang China; ^58^ Qingdao Central Hospital of University of Health and Rehabilitation Sciences, Thoracic Surgery Qingdao China; ^59^ First Affiliated Hospital of Soochow University, Thoracic Surgery Suhou China; ^60^ Changzheng Hospital Affiliated to the Second Military Medical University, Thoracic Surgery Shanghai China; ^61^ The 900th Hospital of Joint Logistic Support Force PLA, Thoracic Surgery Fuzhou China; ^62^ The Southwest Hospital of AMU, Thoracic Surgery Luzhou China; ^63^ PLA General Hospital of Southern Theatre Command, Thoracic Surgery Guangzhou China; ^64^ The First Affiliated Hospital of Nanchang University, Thoracic Surgery Nanchang China; ^65^ Jiaozhou Central Hospital of Qingdao, Thoracic Surgery Qingdao China; ^66^ Ningbo Municipal Second Hospital, Thoracic Surgery Ningbo China; ^67^ Shandong Provincial Hospital, Thoracic Surgery Jinan China; ^68^ Zibo Central Hospital, Thoracic Surgery Zibo China; ^69^ Shanghai Changzheng Hospital, Thoracic Surgery Shanghai China; ^70^ Northern Jiangsu People's Hospital Affiliated to Yangzhou University, Thoracic Surgery Yangzhou China; ^71^ The Affiliated Hospital of Xuzhou Medical University, Thoracic Surgery Xuzhou China; ^72^ The First Affiliated Hospital of Xinjiang Medical University, Thoracic Surgery Urumqi China; ^73^ Linyi Central Hospital, Thoracic Surgery Linyi China; ^74^ Zhejiang University School of Medicine Second Affiliated Hospital, Thoracic Surgery Hangzhou China; ^75^ The Second Affiliated Hospital of Zhengzhou University, Thoracic Surgery Zhengzhou China; ^76^ Cancer Hospital Chinese Academy of Medical Sciences, Thoracic Surgery Beijing China; ^77^ The Third Affiliated Hospital of Chongqing Medical University, Thoracic Surgery Chongqing China; ^78^ Gansu Wuwei Tumour Hospital, Thoracic Surgery Lanzhou China; ^79^ Shaanxi Provincial People's Hospital, Thoracic Surgery Xi'an China; ^80^ Xi'an Chest Hospital, Thoracic Surgery Xi'an China; ^81^ The First Affiliated Hospital of Xi'an Jiaotong University, Thoracic Surgery Xi'an China; ^82^ Yan'an University Xianyang Hospital, Thoracic Surgery Yan'an China; ^83^ Hanzhong Central Hospital, Thoracic Surgery Hanzhong China; ^84^ Shaanxi Provincial Tumor Hospital, Thoracic Surgery Xi'an China; ^85^ First Affiliated Hospital of Xi'an Jiaotong University East Campus, Thoracic Surgery Xi'an China; ^86^ Xi'an Central Hospital, Thoracic Surgery Xi'an China; ^87^ Affiliated Hospital of Shaanxi University of Chinese Medicine, Thoracic Surgery Xianyang China; ^88^ Yulin City First Hospital Yulin Branch, Thoracic Surgery Yulin China; ^89^ Shangluo Central Hospital, Thoracic Surgery Shangluo China; ^90^ Yulin Second Hospital, Thoracic Surgery Yulin China; ^91^ Xi'an Medical University No 2 College, Thoracic Surgery Xi'an China; ^92^ Baoji People's Hospital, Thoracic Surgery Baoji China; ^93^ Xi'an Jiaotong University, Thoracic Surgery Xi'an China; ^94^ Yan'an University Affiliated Hospital, Thoracic Surgery Yan'an China

**Keywords:** expert consensus, subxiphoid and subcostal arch, thymoma, VATS

## Abstract

Surgery is the primary treatment for thymoma. Although subxiphoid and subcostal arch thoracoscopic thymoma surgery is widely used, there is currently a lack of consensus regarding its use, nor have standards been established. Based on the surgical experience of many domestic thoracic surgery centers, the Department of Thoracic Surgery of Tangdu Hospital of Air Force Medical University has formulated this expert consensus regarding key clinical issues related to thoracoscopic thymoma surgery, including preoperative evaluation, surgical indications, preoperative preparation, surgical details, perioperative management, postoperative treatment, and follow‐up. Our aim is to provide consistent and clear guidance for fellow thoracic surgeons to ensure patient safety while optimizing the treatment effect.

## Graphical abstract

Zone 1—from the right side of the midline of the trachea to the inside of the right innominate–internal jugular vein, up to the lower pole of the thyroid gland, down to the upper edge of the left innominate vein, corresponding to the right upper paratracheal group and IASLC2R group of lymph nodes in the deep area of ITMIG; zone 2—from the left side of the midline of the trachea to the inside of the left innominate–internal jugular vein and left common carotid artery, up to the lower pole of the thyroid gland, from the upper edge of the aortic arch, corresponding to the left upper paratracheal group and IASLC2L group of lymph nodes in the ITMIG deep area; zone 4—between the superior vena cava and the aorta, from the lower edge of the left innominate vein to the level of the pericardial reflection at the root of the superior vena cava and the beginning of the ascending aorta, corresponding to the lower paratracheal group and IASLC4 group of lymph nodes in the ITMIG deep area; zone 5—near the lower edge of the innominate vein and the aortic arch, from the lower edge of the left innominate vein to the level of the pulmonary artery trunk, corresponding to the ITMIG deep area subaortic arch/aortopulmonary window group and IASLC5+6 group of lymph nodes; zone 3 is bordered by the right mediastinal pleura, the inner side of the right phrenic nerve, and the outer side of the inner edge of the superior vena cava; zone 6 is delineated by the left mediastinal pleura and the inner side of the left phrenic nerve. Zones 3 and 6 extend from the upper pole of the thoracic cavity to the back of the sternum, the carina, and the front of the superior vena cava and correspond to the ITMIG anterior zone prevascular, para‐aortic, and ascending aorta groups and the IASLC3a group lymph nodes. Zone 7 involves the right cardiophrenic angle area. Zone 8 refers to the pericardial area and zone 9 locates on the left cardiophrenic angle area. Zones 7, 8, and 9 correspond to the ITMIG anterior zone perithymic group, supradiaphragmatic, low phrenic nerve, and pericardial group lymph nodes.

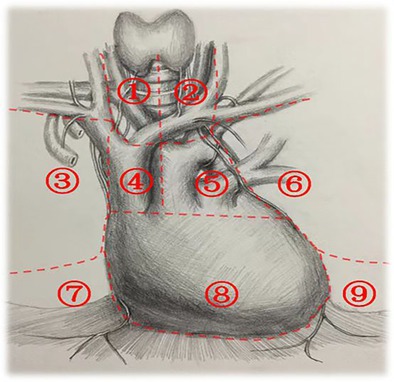

## 1 Introduction and Background

Thymomas are potentially malignant tumors [1]. Their benign or malignant nature is determined not only by histopathology, but also by the presence or absence of capsule infiltration, invasion of surrounding organs, and the presence of distant metastasis [2, 3]. In addition, lymph node status is an important factor in thymoma staging [4, 5, 6]. Surgery is the preferred treatment for thymoma. Even if progression or recurrence occurs after surgery, long‐term survival is possible owing to its relatively indolent nature. Therefore, it is difficult to conduct large‐scale prospective randomized controlled studies regarding the treatment of thymoma [7]. Although thoracoscopic thymoma resection via the subxiphoid and subcostal arch approach is relatively widely used in clinical practice [8, 9, 10], there is no consensus on this procedure in China.

## 2 Selection of Surgical Technique for Thymoma Resection

*Consensus 1*: Based on limited evidence, if minimally invasive surgery can achieve the goals of open surgery, it should be preferentially performed. The recommended minimally invasive technique is thoracoscopic thymomectomy or thymectomy via the subxiphoid and subcostal arch approach, which may be performed several ways: three incisions below the xiphoid process with or without a sternal retractor, a single incision below the xiphoid process, and robot‐assisted using three incisions below the xiphoid process (98.7% agree, 1.3% disagree).

The main goals of surgery are to completely remove the tumor and surrounding adipose tissue, resolve or alleviate clinical symptoms, prevent tumor implantation and metastasis, and avoid surgical complications. Surgeons must also weigh the advantages of the sternotomy approach (wider field of view and higher R0 resection rate) against those of the thoracoscopic approach (more favorable cosmetics, less invasive nature, and less surgery‐related pain). The thoracoscopic approach has become routinely used across China, but many details still need to be optimized and implemented quality control measures [11, 12, 13].

Preoperative diagnosis and examination:

*Consensus 2*: When thymoma is suspected, blood testing for alpha‐fetoprotein and beta‐human chorionic gonadotropin concentrations is recommended to exclude germ cell tumor (97.3% agree, 2.7% disagree).

*Consensus 3*: Contrast‐enhanced magnetic resonance imaging (MRI) of the chest, which can better distinguish thymoma from thymic cysts or thymic hyperplasia, is recommended before surgery as the main means of preoperative staging. This may help to avoid unnecessary surgery. If the patient has a contraindication to contrast administration, non‐contrasted MRI should be obtained (98.1% agree, 1.9% disagree).

*Consensus 4*: Contrast‐enhanced chest computed tomography (CT) is also recommended as a supplementary means of preoperative staging and to determine thymoma resectability. If the patient has a contraindication to contrast administration, non‐contrasted CT should be obtained (99.1% agree, 0.9% disagree).

*Consensus 5*: In patients undergoing contrast‐enhanced chest CT, the contrast medium should be injected into the left upper limb to clearly visualize the left innominate vein (98.2% agree, 1.8% disagree).

Accurate preoperative diagnosis of mediastinal tumors is difficult, and definitive diagnosis requires histopathological examination. However, because thymoma accounts for 50% of primary anterior mediastinal tumors, it should be considered first. For detailed diagnosis and treatment strategies, please refer to the National Comprehensive Care Network (NCCN) guidelines [14]. Alpha‐fetoprotein and β‐human chorionic gonadotropin determination should be performed to exclude germ cell tumor [15, 16]. Contrast‐enhanced MRI of the chest can better distinguish thymoma from thymic cysts and thymic hyperplasia; thus, early imaging identification could avoid unnecessary surgery. In addition, it can enable preoperative staging and help guide surgery [17, 18, 19]. The left innominate vein and the thymus are in close approximation in the mediastinum, and bleeding from unintended vein injury during surgery can be difficult to stop. Therefore, preoperative contrast‐enhanced CT should be performed not only to determine thymoma resectability but also to demonstrate the anatomy of the left innominate vein. This structure is preferentially displayed when the contrast agent is injected into the left upper limb. Non‐contrasted chest CT should be performed in patients with a contraindication to intravenous contrast [20].

## 3 Preoperative Induction Therapy

*Consensus 6*: For stage III and some stage IV thymomas, a multidisciplinary team should evaluate the suitability of surgery. Those considered resectable should undergo surgery, and those that are not should be biopsied (98.4% agree, 1.6% disagree).

*Consensus 7*: For stage III and some stage IV thymomas considered resectable after preoperative induction therapy, a multidisciplinary team should evaluate whether thoracoscopic subcostal thymoma resection is feasible (96.2% agree, 3.8% disagree).

In suspected thymomas, pleural biopsy should be possibly avoided to prevent tumor implantation and metastasis. For stage III and some stage IV thymomas, surgical resection should be performed when possible; biopsy should be considered for those that are not resectable [21]. For tumors initially deemed unresectable via sternotomy, induction therapy is recommended. Once completed, another surgical evaluation should be performed. After surgical resection of the tumor and any isolated metastases, postoperative radiotherapy should be considered. There is insufficient evidence for using the thoracoscopic subcostal approach in patients who have undergone induction therapy. Diagnosis and treatment plans should be developed by a multidisciplinary team comprising radiation oncologists, cardiothoracic surgeons, oncologists, and radiologists.

## 4 Preoperative Preparation for Subxiphoid and Subcostal Arch Thoracoscopic Thymoma Surgery

*Consensus 8*: A comprehensive preoperative assessment of the patient's physical health and medical history and the tumor's characteristics should be performed before formulating a detailed diagnosis and treatment plan (100% agree, 0% disagree).

*Consensus 9*: In patients undergoing resection via the subxiphoid and subcostal arch thoracoscopic approach, intravenous infusion via the left upper limb should be avoided (99% agree, 1% disagree).

Preoperative patient instructions for thoracoscopic thymoma surgery are not different from those for other thoracoscopic surgeries of the chest and mediastinum. Patients with myasthenia gravis must be provided detailed instructions regarding their medication use in the pre‐ and postoperative periods. For perioperative management of patients with other medical problems, the relevant diagnosis and treatment guidelines should be referred to.

Patients with a complex thymoma (locally advanced tumor invasion or isolated metastasis) have special preoperative needs. When a thoracoscopic approach is being used, the surgeon should be prepared to perform open conversion to sternotomy by having the requisite equipment ready and available. Similarly, for tumors invading large blood vessels such as the superior vena cava, the surgeon should be prepared to perform vascular reconstruction. All patients should undergo blood typing and crossmatching in anticipation of needing an intraoperative blood transfusion, as injury to the innominate vein and superior vena cava is possible and the resulting bleeding can be difficult to control. In addition, the transfusion services team should be notified in advance when thoracoscopic thymoma surgery is scheduled. The operating surgeon must evaluate the relationships between the tumor and surrounding organs before surgery and prepare accordingly. Of particular importance is knowledge of what adjacent structures have been invaded by tumor.

When establishing preoperative venous access, two routes should be secured—one for routine fluid infusion and the other for emergency infusion. Superficial venous access should be obtained via the right upper limb because thymoma frequently invades the left innominate vein; therefore, it may be blocked, reconstructed, or removed during surgery. For tumors that invade the innominate vein or other large blood vessels, access via the central veins in the upper limbs should be avoided. If a vascular cutting and closing device is used to remove the innominate vein during surgery, the central vein may be nailed into the stump of the blood vessel, resulting in serious complications such as bleeding, extravasation of infused fluids, and inability to remove the central line. For emergency infusion access, superficial or deep veins in the lower limbs should be used.

The surgical instruments required for thoracoscopic thymoma surgery are as follows ^[8.11–13]^: thoracoscopic equipment, artificial pneumothorax equipment, two 5‐mm operation hole punch cards trocars, two 12‐mm observedation hole punch cards trocars, 30‐cm extended grasping forceps, and a 45‐cm extended ultrasonic scalpel.

## 5 Surgical Process

### 5.1 Surgical Position

*Consensus 10*: The recommended surgical position for subxiphoid and subcostal arch thoracoscopic thymoma surgery is the supine split‐leg position, with the right upper limb abducted 90°. The surgeon stands/sits between the patient's legs, the assistant stands on the patient's right side, and the surgical nurse stands on the patient's left side (95.3% agree, 4.7% disagree).

The skin of the neck, chest, and abdomen should be disinfected from the mandible to the umbilicus between the mid‐axillary lines on both sides. The surgical drapes should fully expose the chest from the sternum to the umbilicus in case conversion to thoracotomy is required. Two positioning details are worth noting: The right upper limb should be abducted to optimize conditions for intravenous infusion; however, not beyond 90°; and the surgeon, assistant, and instrument nurse are in different positions for better coordination [8].

### 5.2 Selection of Surgical Incisions

*Consensus 11*: A 1.5‐ to 3.0‐cm longitudinal incision should be made inferior to the xiphoid process for the observation port. For the operation port, a 0.5‐cm incision is made lateral to the midclavicular line along the costal arch (98.3% agree, 1.7% disagree).

It is advisable to place a trocar in the incision for the observation port here to avoid air leakage during the operation. The subxiphoid incision should not be placed too low to avoid entering the abdominal cavity. After cutting the skin, subcutaneous tissue, fat, and rectus abdominis in sequence, the surgeon must avoid penetrating the peritoneum. Then fingers may be used to probe the linea alba close to the xiphoid process and bluntly separate it cephalad to the prepericardial space to expand space as much as possible. Blunt separation should proceed cephalad for between 3.0 and 5.0 cm. If bleeding occurs, gauze compression should be used for hemostasis. Bleeding from the incisions is rare after surgery. During the separation process, the fingers are attached to the medial side of the costal arch in front and the diaphragm is attached to the attachment surface of the costal arch below, and to the left and right sides approximately to the midline of the bilateral clavicle. For patients with excessive muscle fibers blocking separation to both sides, blunt separation is not suitable. Trocars on both sides can be placed and the ultrasonic knife used for separation. The operation port can be inserted with the index finger placed on the inner side of the sternum to avoid cardiac injury. The operation holes on both sides are as far away from the observation hole as possible to avoid narrow operation space and mutual influence of instruments [12].

### 5.3 Establishment of a Retrosternal Tunnel

*Consensus 12*: Artificial pneumothorax should be induced to expose the surgical space. The recommended pneumothorax pressure setting is 8–14 cm H_2_O (91% agree, 9% disagree).

Once the artificial pneumothorax has been established, gas may leak around the punch card if the incision is too large. If the pressure in the chest cavity becomes insufficient, wet gauze can be used to seal the incision.

Free along the sternal space from both sides and the neck, press the heart downward with the left hand grasping forceps to expose the loose space behind the sternum, open the mediastinal pleura on both sides, and the positive pressure in the chest can effectively press the lungs outward and downward. Grasp the pleura and press it downward lightly, stick to the posterior edge of the sternum, and cut the mediastinal pleura upward until the bilateral internal thoracic veins. Continue to free upward on the inner edge of the vein until the level of the suprasternal fossa (the entrance of the thoracic cavity), with the upper poles of the thymus as the upper limit. The surgical field space is small under the xiphoid costal margin, and the cleanliness of the surgical field is required to be high. Avoiding blunt separation as much as possible helps to ensure that the surgical field of the sternal tunnel is clear and well‐structured, which is conducive to the accurate exposure of anatomical landmarks, but it should not be too close to the sternum. Periosteal injury is also prone to bleeding and contaminating the surgical field.

### 5.4 Urinary Catheterization

*Consensus 13*: If the general anesthesia and operation times are estimated to be less than 2 and 1 h, respectively, and the patient's physical condition allows, a urinary catheter does not need to be placed (81.8% agree, 18.2% disagree).

For experienced thoracic surgeons, the average operation time for a subxiphoid and subcostal arch thoracoscopic thymoma operation is 50 min [8, 12]. However, if pleural adhesions are present or the thymoma is large and predicted to be difficult, a urinary catheter should be placed. Urinary catheterization should also be performed in patients with cardiovascular or cerebrovascular disease and those in whom renal perfusion status should be monitored.

### 5.5 Removal of Specimens and Lymph Nodes

To avoid tumor implantation and metastasis, the tumor‐free principle must be strictly followed when removing specimens and lymph nodes.

### 5.6 Chest Drainage Tube

*Consensus 14*: If no lung tissue resection is performed and the pulmonary pleura is not injured, closed chest tube drainage is not required; however, the chest cavity should be vented before suturing the incisions (91.8% agree, 8.2% disagree).

Thymomectomy and thymectomy are mainly performed within the anterior mediastinum. The integrity of the pleural cavity is basically not damaged. The postoperative pleural inflammatory reaction is small, as is the injury to fat tissue injury. Any pleural effusion generated in reaction to the operation is relatively small. The exudate formed can be rapidly reabsorbed by the pleural cavity. In addition, the blood vessels supplying thymomas are typically small. After hemostasis has been achieved, the probability of postoperative bleeding developing is extremely low. Therefore, routine placement of a chest drainage tube during surgery is not warranted [22]. However, if a pleural effusion is noted during surgery, a drainage tube may be placed. Even when lung tissue is resected during surgery, if no bubbles emerge when the lung is inflated, a drainage tube is not necessarily required. Studies have confirmed that most pleural effusions are eventually naturally absorbed after surgery and that not placing a drainage tube increases patient satisfaction scores and reduces postoperative pain scores. No arc‐shaped liquid density shadows were found in the chest CT scan of the patients 1 month after surgery [23].

## 6 Scope of Surgery

### 6.1 Scope of Thymoma Resection

*Consensus 15*: The scope of resection when performing subxiphoid and subcostal arch thoracoscopic thymoma surgery should consider two factors, tumor stage (both Masaoka–Koga and TNM stages) and the presence of coexisting autoimmune diseases such as myasthenia gravis (99.1% agree, 0.9% disagree).

Controversy exists regarding the appropriate extent of resection in thoracoscopic thymoma surgery. Higher tumor stage and higher degree of pathological malignancy are associated with higher recurrence rates. Although tumor stage, histological type, and resection status are all independent predictors of outcome, the degree of resection is the most important [14, 24, 25].

The NCCN guidelines recommend resection for patients with a resectable thymoma who can tolerate surgery [26, 27, 28, 29]. When planning the scope of resection in thoracoscopic thymectomy, surgeons must consider the Masaoka–Koga and TNM tumor stages and the presence of myasthenia gravis. In addition, they need to decide whether lymph node dissection should be performed, and if so, the extent of lymph node dissection.

*Consensus 16*: For stage I thymoma, extended thymectomy should be performed (96.6% agree, 3.4% disagree).

In the ChART(Chinese thymic Tumor Collaboration Group)study, the 10‐year overall survival rates for thymectomy and thymomectomy were 90.9% and 89.4%, respectively, and the postoperative recurrence rates for stage I tumors were 3.2% and 1.4%, respectively. Both surgical methods seem to be feasible [30, 31, 32, 33, 34]. However, extended thymectomy should be performed for stage I thymoma to ensure adequate resection and avoid unnecessary medical risks.

*Consensus 17*: For stage II thymoma, extended thymectomy should be performed (97.8% agree, 2.2% disagree).

*Consensus 18*: For stage III thymoma, extended thymectomy is required along with removal of any blood vessels invaded by the tumor. Only experienced surgeons should consider using the thoracoscopic approach (97.2% agree, 2.8% disagree).

In patients with resectable stages II and III thymoma, the rate of overall survival is higher and the rate of recurrence is lower with extended thymectomy than with thymomectomy [33, 34]. In the stage II subgroup of the ChART study, the recurrence rate was significantly higher with partial thymectomy than with total thymectomy (14.5% vs. 2.9%). The rates of R1 resection (2.2% vs. 0.7%) and local recurrence (2.1% vs. 0.41%) were significantly higher with thymomectomy than with thymectomy in the Japanese database. It is recommended that extended thymectomy be performed for stage II and III thymomas. Experienced thoracic surgeons can consider extended thymectomy in patients with these tumors using a thoracoscopic approach [12].

*Consensus 19*: For patients with myasthenia gravis, extended thymectomy is recommended. The range of resection is superiorly to the upper poles of the thymus and laterally to the internal thoracic vein on both sides; inferiorly, it proceeds to the cardiophrenic angle and anteriorly to the pericardium, where the lateral extension is 1.0 cm from the medial edge of the phrenic nerve on both sides. All thymus and fat tissue within the above area should be removed (98.3% agree, 1.7% disagree).

For thymoma patients with myasthenia gravis, the Chinese Myasthenia Gravis Guidelines [35], and the American Myasthenia Gravis Association [36, 37] suggest that early surgery can reduce the risk of tumor infiltration and spread. After surgery, the rate of myasthenia gravis remission is higher with extended thymectomy than with thymomectomy. Therefore, complete thymoma resection and extended thymectomy are recommended, mainly because ectopic thymus or aberrant thymus may be present [38, 39]. The thoracoscopic approach is capable of resecting within the recommended range, which is as follows: superiorly to the upper poles of the thymus and laterally to the internal thoracic vein on both sides; inferiorly, it proceeds to the cardiophrenic angle and anteriorly to the pericardium, where the lateral extension is to 1.0 cm from the medial edge of the phrenic nerve on both sides.

### 6.2 Scope of Lymph Node Resection for Thymoma

**FIGURE 1:**
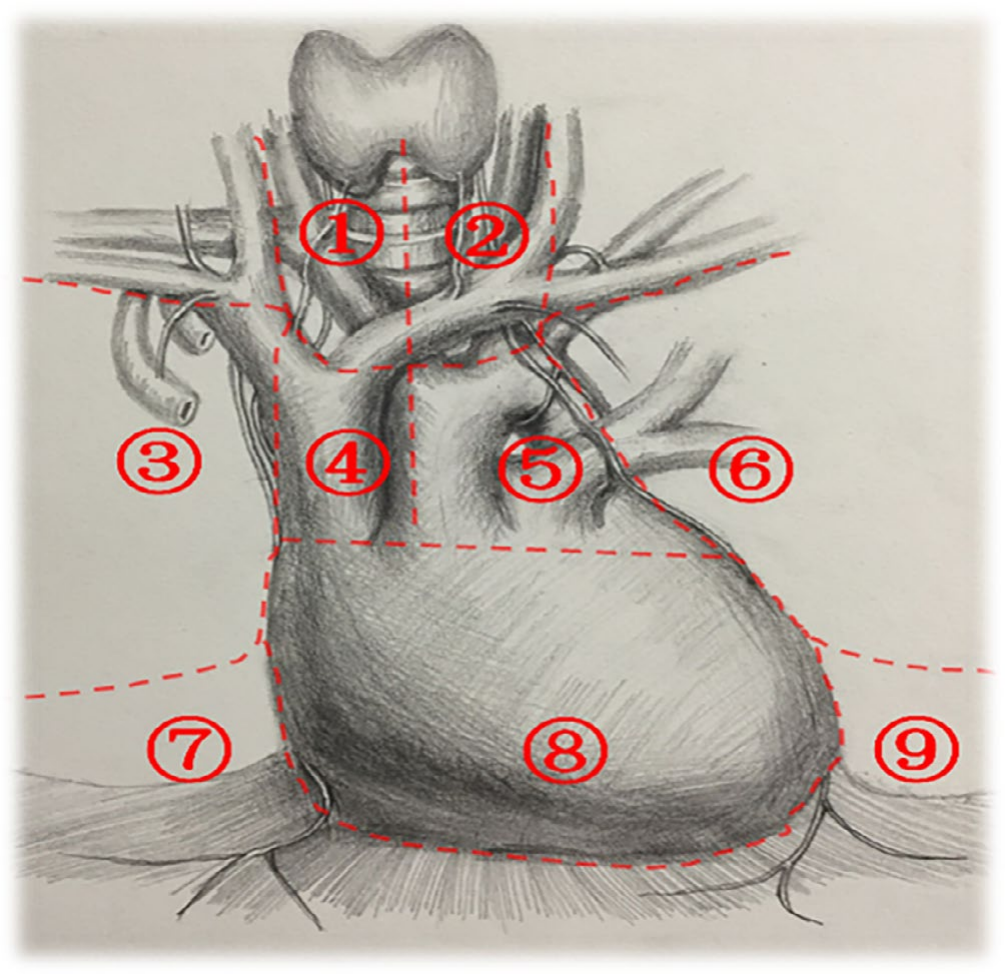
The nine‐zone method.

*Consensus 20*: The rate of lymph node metastasis with thymoma is not negligible. Studies have shown that outcomes are better in patients with negative lymph nodes. Lymph node dissection should be performed using the nine‐zone method recommended by the International Thymic Malignancy Interest Group (ITMIG). The nine‐zone method is shown in Figure 1 (95.5% agree, 4.5% disagree).

*Consensus 21*: For stages I and II thymomas, adjacent and anterior mediastinal lymph nodes should be dissected, which mainly includes the thymic and perithymic lymph nodes (ITMIG N1 regional lymph nodes [zones 3 and 6–9]). The nodes can be removed at the same time as the thymus is removed (94.6% agree, 5.4% disagree).

*Consensus 22*: For stage III thymoma, the ITMIG recommends systematic anterior mediastinal lymph node dissection, as well as systematic lymph node sampling in the paratracheal area and the main pulmonary artery window. All lymph nodes in zones 1–9 should be dissected (91.3% agree, 8.7% disagree).

Thymoma metastasis to lymph nodes is related to histological type and local progression. Although lymph node status is an important factor in patient prognosis, the correlation between thymoma lymph node metastasis and tumor size is inconsistent [40, 41, 42, 43, 44]. The nine zones of lymph node dissection delineated by the ITMIG should be used as a guide, and any nodes suspected to harbor metastasis should be removed [11, 29].

For stages I and II thymomas, dissection of adjacent and anterior mediastinal lymph nodes is recommended. This mainly includes the thymic and perithymic lymph nodes, which correspond to the ITMIG anterior (N1) lymph nodes (zones 3 and 6–9). Zone 3 is bordered by the right mediastinal pleura, the inner side of the right phrenic nerve, and the outer side of the inner edge of the superior vena cava. Zone 6 is delineated by the left mediastinal pleura and the inner side of the left phrenic nerve. Zones 3 and 6 extend from the upper pole of the thoracic cavity to the back of the sternum, the carina, and the front of the superior vena cava and correspond to the ITMIG anterior zone prevascular, para‐aortic, and ascending aorta groups and the IASLC3a group lymph nodes. Zone 7 involves the right cardiophrenic angle area. Zone 8 refers to the pericardial area and zone 9 locates on the left cardiophrenic angle area. Zones 7, 8, and 9 correspond to the ITMIG anterior zone perithymic group, supradiaphragmatic, low phrenic nerve, and pericardial group lymph nodes. The key point is that the lymph nodes can be removed together with the thymus [11, 29, 45].

For stage III thymoma, systematic anterior mediastinal lymph node dissection is recommended, and systematic lymph node sampling is performed in the paratracheal area and the aortopulmonary window. That is, the thoracoscopic approach is used to complete lymph node resection of all locations in zones 1 through 9 as follows: zone 1—from the right side of the midline of the trachea to the inside of the right innominate–internal jugular vein, up to the lower pole of the thyroid gland, down to the upper edge of the left innominate vein, corresponding to the right upper paratracheal group and IASLC2R group of lymph nodes in the deep area of ITMIG; zone 2—from the left side of the midline of the trachea to the inside of the left innominate–internal jugular vein and left common carotid artery, up to the lower pole of the thyroid gland, from the upper edge of the aortic arch, corresponding to the left upper paratracheal group and IASLC2L group of lymph nodes in the ITMIG deep area; zone 4—between the superior vena cava and the aorta, from the lower edge of the left innominate vein to the level of the pericardial reflection at the root of the superior vena cava and the beginning of the ascending aorta, corresponding to the lower paratracheal group and IASLC4 group of lymph nodes in the ITMIG deep area; zone 5—near the lower edge of the innominate vein and the aortic arch, from the lower edge of the left innominate vein to the level of the pulmonary artery trunk, corresponding to the ITMIG deep area subaortic arch/aortopulmonary window group and IASLC5+6 group of lymph nodes [11, 29, 45].

*Consensus 23*: For stage III tumors with local invasion of the pericardium but not the myocardium, or local invasion of the lung, the invaded tissue should be removed (96.5% agree, 3.5% disagree).

*Consensus 24*: For stage III tumors with local invasion of one phrenic nerve in patients without myasthenia gravis who have normal preoperative lung function, the involved phrenic nerve should be removed (88.1% agree, 11.9% disagree).

*Consensus 25*: For stage III tumors with local invasion of the left innominate vein, tumor removal and removal of the left innominate vein with vascular reconstruction should be performed (87.8% agree, 12.2% disagree).

*Consensus 26*: For stage III tumors determined to actually be stage IVA during surgery, the thoracoscopic approach may be continued. A positioning clip should be placed near the tumor margin and any residual lesions to assist with adjuvant radiotherapy (93.2% agree, 6.8% disagree).

The thoracoscopic approach can achieve resection of mediastinal pleura, pericardium, lung, or nerve invaded by tumor. However, when partial resection of invaded tissue is required, the procedure should be performed by a highly experienced thoracoscopic surgeon to minimize impacts on the patient [12].

When left innominate vein resection is required, the risk of postoperative complications is associated with the degree of vein invasion and the surgical method. Preservation of the communicating branches can reduce the incidence of complications related to poor venous return. Given that the thoracoscopic approach is generally associated with preservation of the left internal thoracic vein, pericardial phrenic vein, and communicating branches of the chest wall, and facilitates rapid establishment of collateral circulation, the incidence of complications associated with resection of the left innominate vein is not higher with thoracoscopic resection than with open sternotomy [46, 47, 48].

Patients with stage IV thymoma can undergo emergency surgery. Treatment after surgery can still improve survival and help prevent recurrence [49].

*Consensus 27*: Thymomas should be classified as high or low risk based on the pathological classification and growth pattern determined via histopathological examination of a surgical specimen. Low‐risk tumors comprise T1 thymoma and T2/3 stage type A/AB/B1 thymoma. High‐risk tumors comprise stage T2/3 type B2/B3 thymoma and thymic carcinoma (83% agree, 17% disagree).

*Consensus 28*: Patients with high‐risk thymoma should be examined every 6 months in the first 3 years after surgery, and once a year thereafter for the next 7 years. Those with a low‐risk tumor should be examined every year for 10 years after surgery (95.5% agree, 4.5% disagree).

Based on model analysis, pathological type and tumor stage are independent prognostic factors. These variables are used to classify tumors according to the risk of recurrence. The recurrence rate is significantly lower in T1 thymoma and T2/3 stage type A/AB/B1 thymoma (low‐risk group; 2.7%) than in T2/3 stage type B2/B3 thymoma and thymic carcinoma (high‐risk group; 20.1%; *p* < 0.001). In the high‐risk group, more than half of recurrences (55.2%) occurred within the first 3 years after surgery, and one case recurred 6 years after. In the low‐risk group, the 10‐year recurrence rate was stable [50].

*Consensus 29*: Stage I thymoma in which R0 resection has been achieved does not require adjuvant therapy (100% agree, 0% disagree).

*Consensus 30*: Adjuvant radiotherapy but not chemotherapy is recommended for stage II thymoma in which R0 resection has been achieved (84.8% agree, 15.2% disagree).

*Consensus 31*: Adjuvant radiotherapy, chemotherapy, or both is recommended for stage III thymoma regardless of residual tumor classification. In addition, patients undergoing salvage surgery for stage IV thymoma should receive adjuvant radiotherapy, chemotherapy, or both (100% agree, 0% disagree).

The NCCN and Chinese Anti‐Cancer Association guidelines state that adjuvant therapy is not recommended for completely resected (R0) stage I thymoma. Postoperative radiotherapy can be considered for stage II thymomas after R0 resection, but postoperative chemotherapy is not very meaningful. Currently, these guidelines have clear recommendations for stage I and III thymoma, salvage stage IV thymoma, and thymoma after R1 or R2 resection. However, the evidence that postoperative radiotherapy improves overall survival in stage II thymoma is controversial: some studies indicate that it does while others indicate that it does not. More studies are needed to explore this perspective [51, 52, 53, 54].

## 7 Summary and Outlook

This expert consensus is mainly regarding the subxiphoid and subcostal arch thoracoscopic approach to thymoma. However, many various details require to be further optimized. In addition, few relevant large‐scale multicenter studies have been conducted, which leads to a certain bias. Nonetheless, this consensus has laid a solid foundation for fellow thoracic surgeons to build upon and further develop this procedure. Hopefully, future prospective large‐scale multicenter trials will provide higher‐level evidence to guide treatment.

## Author Contributions

Qiang Lu, Xiaolong Yan, and Yongan Zhou participated in the design of the expert consensus. Xunliang Yin, Hongtao Duan, and Zhengwei Zhao conceived of the expert consensus, participated in its design and other authors coordination, and helped to draft the expert consensus. All authors read and approved the final manuscript.

## Conflicts of Interest

The authors declare no conflicts of interest.

## References

[1] J. Huang , N. P. Rizk , W. D. Travis , et al., “Comparison of Patterns of Relapse in Thymic Carcinoma and Thymoma,” Journal of Thoracic and Cardiovascular Surgery 138, no. 1 (2009): 26–31, 10.1016/j.jtcvs.2009.03.033. 19577051 PMC4151511

[2] A. M. Litvak , K. Woo , S. Hayes , et al., “Clinical Characteristics and Outcomes for Patients With Thymic Carcinoma: Evaluation of Masaoka Staging,” Journal of Thoracic Oncology 9, no. 12 (2014): 1810–1815, 10.1097/JTO.0000000000000363. 25393794 PMC4663074

[3] M. Scorsetti , F. Leo , A. Trama , et al., “Thymoma and Thymic Carcinomas,” Critical Reviews in Oncology/Hematology 99 (2016): 332–350, 10.1016/j.critrevonc.2016.01.012. 26818050

[4] B. Weksler , A. Holden , and J. L. Sullivan , “Impact of Positive Nodal Metastases in Patients With Thymic Carcinoma and Thymic Neuroendocrine Tumors,” Journal of Thoracic Oncology 10, no. 11 (2015): 1642–1647, 10.1097/JTO.0000000000000660. 26317915

[5] B. Weksler , A. Pennathur , J. L. Sullivan , and K. S. Nason , “Resection of Thymoma Should Include Nodal Sampling,” Journal of Thoracic and Cardiovascular Surgery 149, no. 3 (2015): 737–742, 10.1016/j.jtcvs.2014.11.054. 25595379

[6] K. Kondo and Y. Monden , “Lymphogenous and Hematogenous Metastasis of Thymic Epithelial Tumors,” Annals of Thoracic Surgery 76, no. 6 (2003): 1859–1865, 10.1016/s0003-4975(03)01017-8. 14667600

[7] Y. K. Chao , Y. H. Liu , M. J. Hsieh , et al., “Long‐Term Outcomes After Thoracoscopic Resection of Stage I and II Thymoma: A Propensity‐Matched Study,” Annals of Surgical Oncology 22, no. 4 (2015): 1371–1376, 10.1245/s10434-014-4068-9. 25256127

[8] X. Yin , S. Xue , H. Wang , et al., “Clinical Comparative Analyses of Thymectomy Between Subxiphoid and Subcostal Arch Thoracoscopic Resection and Median Sternotomy for the Treatment of Thymoma With Myasthenia Gravis in Chinese Patients,” Journal of Surgical Research 285 (2023): 107–113, 10.1016/j.jss.2022.12.019. 36652769

[9] N. Song , Q. Li , B. Aramini , et al., “Double Sternal Elevation Subxiphoid Versus Uniportal Thoracoscopic Thymectomy Associated With Superior Clearance for Stage I‐II Thymic Epithelial Tumors: Subxiphoid Thymectomy Compared With VATS,” Surgery 172, no. 1 (2022): 371–378, 10.1016/j.surg.2021.12.034. 35164951

[10] H. Xu , D. Liu , Y. Li , et al., “The Outcomes of Subxiphoid Thoracoscopic Versus Video‐Assisted Thoracic Surgery for Thymic Diseases,” Journal of Laparoendoscopic & Advanced Surgical Techniques. Part A 30, no. 5 (2020): 508–513, 10.1089/lap.2019.0734. 32004095

[11] X. Yin , S. Xue , Y. Guo , et al., “Comparative Study of the Clinical Efficacy of Subcostal Thoracoscopy and Median Sternotomy in Treating Thymoma: A Propensity Score‐Matching Analysis,” Journal of International Medical Research 52, no. 1 (2024): 3000605231214470, 10.1177/03000605231214470. 38194488 PMC10777785

[12] J. Zhao , J. Wang , Z. Zhao , et al., “Subxiphoid and Subcostal Arch Thoracoscopic Extended Thymectomy: A Safe and Feasible Minimally Invasive Procedure for Selective Stage III Thymomas,” Journal of Thoracic Disease 8, no. Suppl 3 (2016): S258–S264, 10.3978/j.issn.2072-1439.2016.02.42. 27014472 PMC4783721

[13] Q. Lu , J. Zhao , J. Wang , et al., “Subxiphoid and Subcostal Arch ‘Three Ports’ Thoracoscopic Extended Thymectomy for Myasthenia Gravis,” Journal of Thoracic Disease 10, no. 3 (2018): 1711–1720, 10.21037/jtd.2018.02.11. 29707325 PMC5906355

[14] F. C. Detterbeck and A. Zeeshan , “Thymoma: Current Diagnosis and Treatment,” Chinese Medical Journal 126, no. 11 (2013): 2186–2191. 23769581

[15] A. Marx , J. K. Chan , J. M. Coindre , et al., “The 2015 World Health Organization Classification of Tumors of the Thymus: Continuity and Changes,” Journal of Thoracic Oncology 10, no. 10 (2015): 1383–1395, 10.1097/JTO.0000000000000654. 26295375 PMC4581965

[16] A. Marx , P. Ströbel , S. S. Badve , et al., “ITMIG Consensus Statement on the Use of the WHO Histological Classification of Thymoma and Thymic Carcinoma: Refined Definitions, Histological Criteria, and Reporting,” Journal of Thoracic Oncology 9, no. 5 (2014): 596–611, 10.1097/JTO.0000000000000154. 24722150

[17] T. T. M. Thuy , N. T. H. Trang , T. T. Vy , et al., “Role of Diffusion‐Weighted MRI in Differentiation Between Benign and Malignant Anterior Mediastinal Masses,” Frontiers in Oncology 12 (2022): 985735, 10.3389/fonc.2022.985735. 36313699 PMC9606681

[18] X. Zhang , R. Zhang , Y. Cao , X. Wang , and Y. Chen , “The Value of Enhanced Computed Tomography Combined With Magnetic Resonance Imaging in the Differential Diagnosis of Thymomas and Thymic Cysts Before Operation,” Translational Cancer Research 10, no. 6 (2021): 2777–2789, 10.21037/tcr-21-96. 35116588 PMC8799001

[19] E. M. Marom , M. L. Rosado‐de‐Christenson , J. F. Bruzzi , M. Hara , J. R. Sonett , and L. Ketai , “Standard Report Terms for Chest Computed Tomography Reports of Anterior Mediastinal Masses Suspicious for Thymoma,” Journal of Thoracic Oncology 6, no. 7 Suppl 3 (2011): S1717–S1723, 10.1097/JTO.0b013e31821e8cd6. 21847053

[20] J. Sadohara , K. Fujimoto , N. L. Müller , et al., “Thymic Epithelial Tumors: Comparison of CT and MR Imaging Findings of Low‐Risk Thymomas, High‐Risk Thymomas, and Thymic Carcinomas,” European Journal of Radiology 60, no. 1 (2006): 70–79, 10.1016/j.ejrad.2006.05.003. 16766154

[21] T. Araki , M. Nishino , W. Gao , et al., “Anterior Mediastinal Masses in the Framingham Heart Study: Prevalence and CT Image Characteristics,” European Journal of Radiology Open 2 (2015): 26–31, 10.1016/j.ejro.2014.12.003. 25705709 PMC4332399

[22] N. M. Rahman , J. Pepperell , S. Rehal , et al., “Effect of Opioids vs NSAIDs and Larger vs Smaller Chest Tube Size on Pain Control and Pleurodesis Efficacy Among Patients With Malignant Pleural Effusion: The TIME1 Randomized Clinical Trial,” JAMA 315, no. 7 (2016): 707, 10.1001/jama.2016.0428. 26720026

[23] T. Zhang , Z. Zhao , Y. Zhou , et al., “Safety Study of Thoracic Drainage Tube Without Indentation After Excision of Anterior Mediastinal Lesions Under Xiphoid Costal Margin,” Chinese Journal of Thoracic and Cardiovascular Surgery 36, no. 11 (2020): 668–671, 10.3760/cma.j.cn112434-20200921-00430.

[24] F. C. Detterbeck and A. M. Parsons , “Management of Stage I and II Thymoma,” Thoracic Surgery Clinics 21, no. 1 (2011): 59–vii, 10.1016/j.thorsurg.2010.08.001. 21070987

[25] F. Detterbeck , S. Youssef , E. Ruffini , and M. Okumura , “A Review of Prognostic Factors in Thymic Malignancies,” Journal of Thoracic Oncology 6, no. 7 Suppl 3 (2011): S1698–S1704, 10.1097/JTO.0b013e31821e7b12. 21847050

[26] A. Masaoka , “Staging System of Thymoma,” Journal of Thoracic Oncology 5, no. 10 Suppl 4 (2010): S304–S312, 10.1097/JTO.0b013e3181f20c05. 20859124

[27] J. E. Lewis , M. R. Wick , B. W. Scheithauer , P. E. Bernatz , and W. F. Taylor , “Thymoma. A Clinicopathologic Review,” Cancer 60, no. 11 (1987): 2727–2743, 10.1002/1097-0142(19871201)60:11<2727::aid-cncr2820601125>3.0.co;2-d. 3677008

[28] M. F. Benveniste , R. J. Korst , A. Rajan , F. C. Detterbeck , E. M. Marom , and International Thymic Malignancy Interest Group , “A Practical Guide From the International Thymic Malignancy Interest Group (ITMIG) Regarding the Radiographic Assessment of Treatment Response of Thymic Epithelial Tumors Using Modified RECIST Criteria,” Journal of Thoracic Oncology 9, no. 9 Suppl 2 (2014): S119–S124, 10.1097/JTO.0000000000000296. 25396308

[29] Y. Hwang , I. K. Park , S. Park , E. R. Kim , C. H. Kang , and Y. T. Kim , “Lymph Node Dissection in Thymic Malignancies: Implication of the ITMIG Lymph Node Map, TNM Stage Classification, and Recommendations,” Journal of Thoracic Oncology 11, no. 1 (2016): 108–114, 10.1016/j.jtho.2015.09.001. 26762745

[30] T. Onuki , S. Ishikawa , K. Iguchi , et al., “Limited Thymectomy for Stage I or II Thymomas,” Lung Cancer 68, no. 3 (2010): 460–465, 10.1016/j.lungcan.2009.08.001. 19717204

[31] M. K. Bae , S. K. Lee , H. Y. Kim , et al., “Recurrence After Thymoma Resection According to the Extent of the Resection,” Journal of Cardiothoracic Surgery 9 (2014): 51, 10.1186/1749-8090-9-51. 24646138 PMC3994658

[32] A. Fiorelli , G. Natale , C. Freda , and M. Santini , “Is Thymomectomy Equivalent to Complete Thymectomy in Non‐Myasthenic Patients With Early‐Stage Thymoma?,” Interactive Cardiovascular and Thoracic Surgery 28, no. 3 (2019): 399–403. 30188996 10.1093/icvts/ivy270

[33] Z. Gu , J. Fu , Y. Shen , et al., “Thymectomy Versus Tumor Resection for Early‐Stage Thymic Malignancies: A Chinese Alliance for Research in Thymomas Retrospective Database Analysis,” Journal of Thoracic Disease 8 (2016): 680–686. 27114835 10.21037/jtd.2016.03.16 PMC4824726

[34] K. Nakagawa , K. Yokoi , J. Nakajima , et al., “Is Thymomectomy Alone Appropriate for Stage I (T1N0M0) Thymoma?Results of a Propensity‐Score Analysis,” Annals of Thoracic Surgery 101 (2016): 520–526. 26482784 10.1016/j.athoracsur.2015.07.084

[35] T. Chang , “Chinese Guidelines for Diagnosis and Treatment of Myasthenia Gravis (2020 Edition),” Chinese Journal of Neuroimmunology and Neurology 28, no. 1 (2021): 1–12.

[36] P. Narayanaswami , D. B. Sanders , G. Wolfe , et al., “International Consensus Guidance for Management of Myasthenia Gravis: 2020 Update,” Neurology 96, no. 3 (2021): 114–122, 10.1212/WNL.0000000000011124. 33144515 PMC7884987

[37] D. B. Sanders , G. I. Wolfe , M. Benatar , et al., “International Consensus Guidance for Management of Myasthenia Gravis: Executive Summary,” Neurology 87, no. 4 (2016): 419–425, 10.1212/WNL.0000000000002790. 27358333 PMC4977114

[38] A. Jaretzki, 3rd , A. S. Penn , D. S. Younger , et al., “Maximal Thymectomy for Myasthenia Gravis. Results,” Journal of Thoracic and Cardiovascular Surgery 95, no. 5 (1988): 747–757. 3361927

[39] M. Zielinski , L. Hauer , J. Hauer , J. Pankowski , T. Nabialek , and A. Szlubowski , “Comparison of Complete Remission Rates After 5 Year Follow‐Up of Three Different Techniques of Thymectomy for Myasthenia Gravis,” European Journal of Cardio‐Thoracic Surgery 37, no. 5 (2010): 1137–1143, 10.1016/j.ejcts.2009.11.029. 20117014

[40] Z. Gu , Y. Wei , J. Fu , et al., “Lymph Node Metastases in Thymic Malignancies: A Chinese Alliance for Research in Thymomas Retrospective Database Analysis,” Interactive Cardiovascular and Thoracic Surgery 25, no. 3 (2017): 455–461, 10.1093/icvts/ivx116. 28521033

[41] W. Fang , Y. Wang , L. Pang , et al., “Lymph Node Metastasis in Thymic Malignancies: A Chinese Multicenter Prospective Observational Study,” Journal of Thoracic and Cardiovascular Surgery 156, no. 2 (2018): 824–833.e1, 10.1016/j.jtcvs.2018.04.049. 29778330

[42] I. K. Park , Y. T. Kim , J. H. Jeon , et al., “Importance of Lymph Node Dissection in Thymic Carcinoma,” Annals of Thoracic Surgery 96, no. 3 (2013): 1025–1032, 10.1016/j.athoracsur.2013.04.057. 23806232

[43] H. Clermidy , J. M. Maury , S. Collaud , et al., “Lymph Node Dissection in Thymoma: Is It Worth It?,” Lung Cancer 157 (2021): 156–162, 10.1016/j.lungcan.2021.05.022. 34053783

[44] U. Ahmad and S. Raja , “Lymph Node Metastases in Thymic Tumors: The More We Know, the Less We Know,” Journal of Thoracic and Cardiovascular Surgery 154, no. 1 (2017): e15–e16, 10.1016/j.jtcvs.2017.03.108. 28457540

[45] V. W. Rusch , H. Asamura , H. Watanabe , D. J. Giroux , R. Rami‐Porta , and P. Goldstraw , “The IASLC Lung Cancer Staging Project: A Proposal for a New International Lymph Node Map in the Forthcoming Seventh Edition of the TNM Classification for Lung Cancer,” Journal of Thoracic Oncology 4, no. 5 (2009): 568–577, 10.1097/JTO.0b013e3181a0d82e. 19357537

[46] C. B. Sai Sudhakar and J. A. Elefteriades , “Safety of Left Innominate Vein Division During Aortic Arch Surgery,” Annals of Thoracic Surgery 70, no. 3 (2000): 856–858, 10.1016/s0003-4975(00)01498-3. 11016323

[47] A. McPhee , K. Shaikhrezai , and G. Berg , “Is It Safe to Divide and Ligate the Left Innominate Vein in Complex Cardiothoracic Surgeries?,” Interactive Cardiovascular and Thoracic Surgery 17, no. 3 (2013): 560–563, 10.1093/icvts/ivt244. 23736661 PMC3745152

[48] H. Q. Wang , F. Tian , M. Wei , et al., “Preliminary Evaluation and Discussion of the Safety of Left Innominate Vein Resection,” Journal of Thoracic Disease 12, no. 3 (2020): 438–447, 10.21037/jtd.2020.01.29. 32274110 PMC7139071

[49] E. Ruffini and F. Venuta , “Management of Thymic Tumors: A European Perspective,” Journal of Thoracic Disease 6, no. Suppl 2 (2014): S228–S237, 10.3978/j.issn.2072-1439.2014.04.19. 24868441 PMC4032964

[50] H. Liu , Z. Gu , B. Qiu , et al., “A Recurrence Predictive Model for Thymic Tumors and Its Implication for Postoperative Management: A Chinese Alliance for Research in Thymomas Database Study,” Journal of Thoracic Oncology 15, no. 3 (2020): 448–456, 10.1016/j.jtho.2019.10.018. 31726106

[51] T. Utsumi , H. Shiono , Y. Kadota , et al., “Postoperative Radiation Therapy After Complete Resection of Thymoma Has Little Impact on Survival,” Cancer 115, no. 23 (2009): 5413–5420, 10.1002/cncr.24618. 19685527

[52] A. A. Mangi , C. D. Wright , J. S. Allan , et al., “Adjuvant Radiation Therapy for Stage II Thymoma,” Annals of Thoracic Surgery 74, no. 4 (2002): 1033–1037, 10.1016/s0003-4975(02)03828-6. 12400741

[53] M. Myojin , N. C. Choi , C. D. Wright , et al., “Stage III Thymoma: Pattern of Failure After Surgery and Postoperative Radiotherapy and Its Implication for Future Study,” International Journal of Radiation Oncology, Biology, Physics 46, no. 4 (2000): 927–933, 10.1016/s0360-3016(99)00514-3. 10705015

[54] Y. J. Lim , H. J. Kim , and H. G. Wu , “Role of Postoperative Radiotherapy in Nonlocalized Thymoma: Propensity‐Matched Analysis of Surveillance, Epidemiology, and End Results Database,” Journal of Thoracic Oncology 10, no. 9 (2015): 1357–1363. 26280586 10.1097/JTO.0000000000000619

